# Variation in *TMEM106B* in chronic traumatic encephalopathy

**DOI:** 10.1186/s40478-018-0619-9

**Published:** 2018-11-04

**Authors:** Jonathan D. Cherry, Jesse Mez, John F. Crary, Yorghos Tripodis, Victor E. Alvarez, Ian Mahar, Bertrand R. Huber, Michael L. Alosco, Raymond Nicks, Bobak Abdolmohammadi, Patrick T. Kiernan, Laney Evers, Sarah Svirsky, Katharine Babcock, Hannah M. Gardner, Gaoyuan Meng, Christopher J. Nowinski, Brett M. Martin, Brigid Dwyer, Neil W. Kowall, Robert C. Cantu, Lee E. Goldstein, Douglas I. Katz, Robert A. Stern, Lindsay A. Farrer, Ann C. McKee, Thor D. Stein

**Affiliations:** 1 Boston University Alzheimer’s Disease and CTE Center, Boston University School of Medicine, 72 E Concord Street, B7800, Boston, MA 02118 USA; 2 Department of Neurology, Boston University School of Medicine, 72 E Concord Street, B7800, Boston, MA 20118 USA; 3 VA Boston Healthcare System, 150 S. Huntington Avenue, Boston, MA 02130 USA; 4 Department of Biostatistics, Boston University School of Public Health, Boston, MA 20118 USA; 5 Department of Veterans Affairs Medical Center, Bedford, MA 01730 USA; 6 Department of Pathology, Fishberg Department of Neuroscience, Friedman Brain Institute, Ronald M. Loeb Center for Alzheimer’s Disease, Icahn School of Medicine at Mount Sinai School, New York, NY 10029 USA; 7 Department of Anatomy and Neurobiology, Boston University School of Medicine, Boston, MA 20119 USA; 8 Concussion Legacy Foundation, Boston, MA 02115 USA; 9 Department of Neurosurgery, Boston University School of Medicine, 72 E Concord Street, B7800, Boston, MA 02118 USA; 10 Department of Neurosurgery, Emerson Hospital, Concord, MA 01742 USA; 11 Molecular Aging and Development Laboratory, Boston University School of Medicine, 72 E Concord Street, B7800, Boston, MA 02118 USA; 12 Boston University Photonics Center, Boston University, Boston, MA 02118 USA; 13 College of Engineering, Boston University, Boston, MA USA; 14 Braintree Rehabilitation Hospital, Braintree, MA 02118 USA; 15 Department of Pathology and Laboratory Medicine, Boston University School of Medicine, 72 E Concord Street, B7800, Boston, MA 02118 USA; 16 Department of Medicine (Biomedical Genetics), Boston University School of Medicine, 72 E Concord Street, B7800, Boston, MA 02118 USA; 17 Department of Ophthalmology, Boston University School of Medicine, 72 E Concord Street, B7800, Boston, MA 02118 USA; 18 Department of Epidemiology, Boston University School of Public Health, Boston, MA 02118 USA; 19 Midwestern University Arizona College of Osteopathic Medicine, Glendale, AZ 85308 USA

**Keywords:** Chronic traumatic encephalopathy, TMEM106B, Neuroinflammation, Football, Traumatic brain injury, Tau, Genetics, TDP-43, Dementia

## Abstract

The genetic basis of chronic traumatic encephalopathy (CTE) is poorly understood. Variation in transmembrane protein 106B (*TMEM106B*) has been associated with enhanced neuroinflammation during aging and with TDP-43-related neurodegenerative disease, and rs3173615, a missense coding SNP in *TMEM106B*, has been implicated as a functional variant in these processes. Neuroinflammation and TDP-43 pathology are prominent features in CTE. The purpose of this study was to determine whether genetic variation in *TMEM106B* is associated with CTE risk, pathological features, and ante-mortem dementia. Eighty-six deceased male athletes with a history of participation in American football, informant-reported Caucasian, and a positive postmortem diagnosis of CTE without comorbid neurodegenerative disease were genotyped for rs3173615*.* The minor allele frequency (MAF = 0.42) in participants with CTE did not differ from previously reported neurologically normal controls (MAF = 0.43). However, in a case-only analysis among CTE cases, the minor allele was associated with reduced phosphorylated tau (ptau) pathology in the dorsolateral frontal cortex (DLFC) (AT8 density, odds ratio [OR] of increasing one quartile = 0.42, 95% confidence interval [CI] 0.22–0.79, *p* = 0.008), reduced neuroinflammation in the DLFC (CD68 density, OR of increasing one quartile = 0.53, 95% CI 0.29–0.98, *p* = 0.043), and increased synaptic protein density (β = 0.306, 95% CI 0.065–0.546, *p* = 0.014). Among CTE cases, *TMEM106B* minor allele was also associated with reduced ante-mortem dementia (OR = 0.40, 95% CI 0.16–0.99, *p* = 0.048), but was not associated with TDP-43 pathology. All case-only models were adjusted for age at death and duration of football play. Taken together, variation in *TMEM106B* may have a protective effect on CTE-related outcomes.

## Introduction

Chronic traumatic encephalopathy (CTE) is a progressive neurodegenerative disease that has been neuropathologically diagnosed in individuals with a history of repetitive head impacts (RHI) [22], including contact and collision sport athletes who participated in American football, ice hockey, rugby, mixed martial arts, soccer, and boxing [20]. Currently, CTE can only be diagnosed post-mortem. In a recent report describing a convenience sample of 202 former American football players, 99% of former National Football League (NFL) players were neuropathologically diagnosed with CTE at autopsy. Although the frequency of CTE in individuals with less football exposure was substantial, it was nonetheless lower (highest level of play - college: 91%; highest level of play – high school: 21%) [24]. Further, among those with CTE, former college and professional players had both mild and severe CTE pathology. It is unclear why among players with comparable RHI exposure, only some develop CTE or why disease severity varies. This variation may be due to individual differences in genetic, demographic, athletic or comorbid pathologic factors [1, 17, 39]. Previous small studies have identified suggestive relationships between CTE and candidate genetic factors such as apolipoprotein E4 (*APOE* ϵ4), microtubule associated protein tau (*MAPT*), and transmembrane protein 106b (*TMEM106B*) [3]. However, these relationships were not statistically significant and/or have not been replicated [21, 38].

Several converging lines of evidence suggest that *TMEM106B* may be involved in the development of CTE. In CTE, previous studies have implicated neuroinflammation as a potential disease mechanism, with chronic microglial activation triggering a positive feedback loop with the hyperphosphorylation and aggregation of tau [10]. *TMEM106B* may have a protective role with respect to neuroinflammation. A recent genome wide associate study (GWAS) of age-associated transcription changes implicated *TMEM106B* and found that the effect size was amplified when limited to a microglia-associated gene expression cluster [34]. The top-ranked single nucleotide polymorphism (SNP), rs1990622, was associated with a transcription pattern in the frontal cortex linked to younger chronological age. Among participants greater than age 65, the rs1990622 minor allele was associated with improved cognitive status. In human monocyte-derived dendritic cells, rs1990622 was associated with a reduced inflammatory state. Additionally, variation in *TMEM106B* is associated with TDP-43 pathology [43], a feature found in many cases of CTE [21], as well as several other neurodegenerative diseases. In a GWAS of frontotemporal lobar degeneration (FTLD)-TDP, rs1990622 was the top SNP and achieved genome wide significance [43]. Rs1990622 also has been associated with cognitive impairment in amyotrophic lateral sclerosis (ALS) [44], hippocampal sclerosis in normal aging [28], and incidence of Alzheimer’s disease (AD) in *APOE* ϵ4 negative individuals [14, 36].

Here, we assessed rs3173615, the only coding SNP in high linkage disequilibrium (LD) with rs1990622, for association with CTE-related outcomes. Rs3173615 encodes a change from threonine to serine at position 185 (p.T185S), may regulate TMEM106B protein levels, and is a suggested functional variant underlying the association with FTLD-TDP [30]. We hypothesized that the minor allele of rs3173615 is associated with CTE risk and, among those with CTE, with reduced tau aggregation, TDP-43 burden, neuroinflammation, synaptic loss and ante-mortem dementia.

## Materials and methods

### Participant selection

**Fig. 1:**
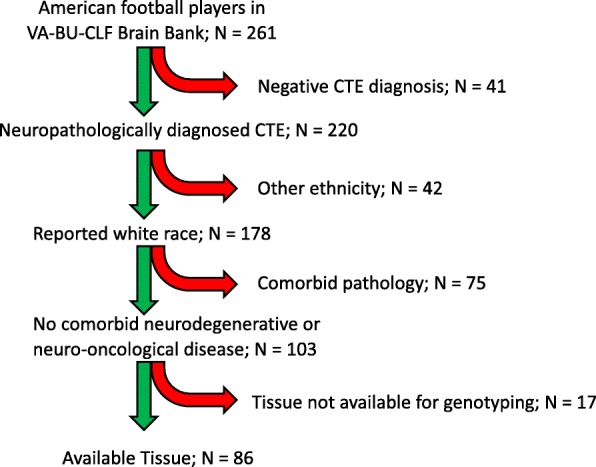
Inclusion flow chart for CTE Cases. Red curved arrows indicate participants that were excluded. Tissue was not available for genotyping for the following reasons: 1) consults with tissue returned to consulting neuropathologist; 2) only small fragments received; 3) tissue was significantly degraded and did not pass quality control for genotyping

All CTE cases were ascertained from the Veterans Affairs-Boston University-Concussion Legacy Foundation (VA-BU-CLF) Brain Bank. Details on inclusion criteria have been described previously [26]. To be eligible for the brain bank, participants needed to have exposure to RHI, either from contact sports, military service or domestic violence. Clinical symptoms were not considered in the inclusion criteria. Participants of the current study were restricted to those who played American football, who were reported to be Caucasian, and who were neuropathologically diagnosed with CTE without significant co-morbid neurodegenerative disease. Because *TMEM106B* has been implicated in several other neurodegenerations many of which can occur comorbid with CTE [39, 46], we excluded participants with other significant co-morbid neurodegenerative disease. A total of 86 of 261 brain bank participants met these criteria (Fig. 1). An authorized legal representative provided written consent for participation and brain donation. IRB approval for the brain donation program was obtained through the Boston University Alzheimer’s Disease & CTE Center and the Edith Nourse Rogers Memorial Veterans Hospital. Neurologically normal controls were ascertained from a previous study of frontotemporal dementia [42]. These 376 participants had undergone genotyping at rs3173615 and were found to have no evidence of cognitive impairment or motor neuron disease on clinical assessment.

### Neuropathological assessment

VA-BU-CLF Brain Bank methods for pathologic processing of tissue and neuropathologic evaluation have been detailed elsewhere [45]. Briefly, the following stains were used: luxol fast blue, hematoxylin and eosin, Bielschowsky’s silver, hyperphosphorylated tau (p-tau) (AT8), alpha synuclein, amyloid beta (Aβ), and phosphorylated TDP-43 using previously published methods [18, 19, 22]. TDP-43 pathology was assessed in paraffin sections from the dorsolateral frontal cortex (DLFC), hippocampus, amygdala, entorhinal cortex, and midbrain. The neuropathological diagnosis of CTE was made using the National Institute of Neurological Disorders and Stroke (NINDS)/ National Institute of Biomedical Imaging and Bioengineering (NIBIB) consensus criteria based on the presence of abnormal perivascular accumulations of p-tau in neurons, astrocytes, and cell processes in an irregular and patchy distribution concentrated at the depths of cortical sulci [18]. Other neurodegenerative diseases were diagnosed using well-established criteria for AD, dementia with Lewy bodies (DLB), FTLD, and motor neuron disease (MND) [4, 6, 7, 12, 15, 16, 23, 27, 29]. Neuropathologists were blinded to all clinical data at the time of diagnosis.

### Clinical assessment

VA-BU-CLF Brain Bank clinical assessment was conducted as previously described utilizing telephone clinical interviews with informants, online questionnaires, and medical record review [26, 38, 40]. Briefly, each of the following was assessed: type and amount (in years) of contact sport play, military service, whether mood, behavioral or cognitive symptoms were present and progressed over time, presence of functional impairment, and cause of death. Clinical history was presented at a multidisciplinary clinical consensus conference where it was determined whether the participant met criteria for dementia [26]. All interviews were conducted blinded to the results of the neuropathological examination.

### Genotyping

For CTE cases, genomic DNA was extracted from cerebellum using the Qiagen DNeasy Blood & Tissue Kit. Genotyping of rs3173615 was performed using iPLEX Assay and MassARRAY System as per manufactures protocols. Controls were also genotyped using an iPLEX Assay and MassARRAY System, but not as part of the same batch.

### Enzyme-linked immunosorbent assay (ELISA)

Flash frozen brain tissue was obtained from 37 of the 86 CTE cases as previously described [9]. Frozen tissue was collected from identical regions in Broadman area 8/9. Briefly, freshly prepared, ice cold 5 M Guanidine Hydrochloride in Tris-buffered saline (20 mM Tris-HCl, 150 mM NaCl, pH 7.4 TBS) containing 1:100 Halt protease inhibitor cocktail (Thermo Scientific) and 1:100 Phosphatase inhibitor cocktail 2 & 3 (Sigma) was added to the brain tissue at 5:1 and homogenized with Qiagen Tissue Lyser LT, at 50 Hz for 5 min. Lysate was diluted according to manufacture protocol and spun down at 17,000 g, 4 °C, for 15 min. Supernatant was investigated using a PSD-95 ELISA (MSD #K250QND) and run according to manufactures protocols. Plates were analyzed with an MSD SECTOR S 600 Imager, and results were reported as arbitrary values. Values appeared normally distributed on visual inspection and then were converted to z-scores with a mean of zero and standard deviation of one.

### Digital histology and analysis

Immunostaining for AT8 and CD68 and analysis using the Aperio ScanScope (Leica) were performed as previously described [10]. Briefly, tissue blocks of cortical samples were taken from Broadman area 8/9 for all cases. Whole stained DLFC sections were scanned and digitized using an Aperio ScanScope AT Turbo. Digital images were viewed and analyzed using Aperio ImageScope (Leica). Analysis of digital images were limited to the depth of the superior frontal sulcus which was denoted as the bottom third of the connecting superior and middle gyri. White matter was excluded. A customized version of the Aperio positive pixel count algorithm (Version 9) was used to determine total AT8 positive staining. Similarly, a modified nuclear count algorithm (Version 9) was used to count total number of CD68 positive cells. Densities in units of count per mm^2^ were obtained by standardizing quantifications to the area measured. For both AT8-positive pixel density and CD68-positive cell density, participants were stratified into quartiles to account for the rightward skew of the densities.

### Statistical analysis

The association of rs3173615 with six dimensions of CTE-related outcomes (presence of positive CTE neuropathological diagnosis, AT8-positive pixel density in the DLFC, CD68-positive cell density in the DLFC, synaptic density as measured by PSD-95 ELISA, presence of TDP-43 pathology in any brain region and ante-mortem dementia) was evaluated using an additive genetic model. Genotype and allele frequencies were compared between cases and controls using the Cochrane-Armitage Trend test and chi-squared test respectively. All other analyses were only conducted among those with CTE (case-only analyses). We used separate ordinal logistic regression models to estimate the relative odds of a one quartile increase in AT8-positive pixel density or CD68-positive cell density for each additional minor allele, linear regression to estimate differences in PSD-95 synaptic density for each additional minor allele, and separate binary logistic regression models to estimate the relative of odds of having anti-mortem dementia or having TDP-43 pathology for each additional minor allele. All case-only analyses were adjusted for age at death and years of football play. Statistical analyses were performed using SPSS (v.24, IBM) and R (v.3.5.0).

## Results

### *TMEM106B* genotype is not associated with CTE diagnosis

Clinical and pathological characteristics of the participants with CTE are presented in Table 1. Controls were significantly older than the CTE cases (*p* = 0.01). Rs3173615 allele and genotype frequencies were not significantly different between cases and controls (*p* = 0.71 and *p* = 0.74, respectively).

**Table 1:** Clinical, genetic and pathologic characteristics of participants

	Controls (*n* = 376)	CTE cases (*n* = 86)	*P* Value
Age (mean ± SD (range))	61.02 ± 10.2 (35–90)	57.02 ± 21.19 (17–89)	0.01
Years of exposure (mean ± SD (range))	–	13.49 ± 5.47 (1–31)	–
Cases with dementia (%)	–	33 (38.4%)	–
*TMEM106B* MAF	43.2%	41.9%	0.71
rs3173615 genotypes			0.74
CC (%)	123 (32.7%)	29 (33.7%)	
CG (%)	181 (48.1%)	42 (48.8%)	
GG (%)	72 (19.1%)	15 (17.4%)	
CERAD (mean ± SD)	–	0.29 ± 0.50	–
Cases with TDP-43 (%)	–	27 (31.4%)	–
AT8 Quartiles (positive pixel density/mm^2^ ± SD)			
1	–	283 ± 221	
2	–	2863 ± 1362	
3	–	10,438 ± 3715	
4	–	65,914 ± 62,629	
CD68 Quartiles (Positive cell/mm^2^ ± SD)			
1	–	93 ± 15	
2	–	138 ± 10	
3	–	171 ± 10	
4	–	226 ± 31	

*CERAD* Consortium to establish a registry for Alzheimer’s disease, *CTE* Chronic Traumatic Encephalopathy (Ranges from Stage I-IV), *MAF* minor allele frequency

### *TMEM106B* genotype is associated with CTE-related neuropathology and ante-mortem dementia in persons with CTE

**Fig. 2:**
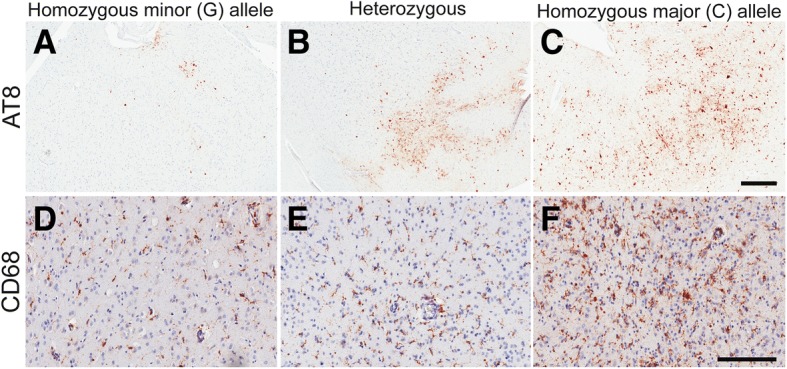
Representative images of ptau (AT8) and neuroinflammation (CD68) staining by *TMEM106B* genotype. Positive staining for the respective proteins is in red while hematoxylin counterstain is blue. All images are from the DLFC at the depth of the cortical sulcus. Scale bars represent 500 μm (**a–c**) and 200 μm (**d–f**)

The rs3173615 minor allele (G) was significantly associated with lower p-tau (AT8-positive) pixel density in the DLFC in a dose-dependent manner: for each G allele, the odds of increasing one quartile in p-tau pixel density was 0.42 (Fig. 2, Table 2). The G allele was significantly associated with lower CD68-positive cell density in the DLFC in a dose-dependent manner: for each G allele, the odds of increasing one quartile in CD68-positive cell density was 0.53 (Fig. 2, Table 2). The G allele was significantly associated with higher synaptic density, as measured by PSD-95 ELSIA, in a dose-dependent manner: for each G allele, synaptic density increased by 0.31 standardized units (Table 2). Rs3173615 genotype was not associated with the presence of TDP-43 pathology. However, the presence of each G allele reduced odds of dementia prior to death by 60% (Table 3).

**Table 2:** Ordinal and linear regression models predicting AT8 tau deposition, CD68 cell density, and PSD-95 concentration

	AT8 tau pathology			CD68 cell density			PSD-95 concentration		
	OR	95% CI	*p*-value	OR	95% CI	*p*-value	B	95% CI	*p*-value
*TMEM106B* minor G allele (additive)	0.42	0.22–0.79	0.008	0.53	0.29–0.98	0.04	0.31	0.07–0.55	0.01

*TMEM106B* genotype *rs3173615*; additive genetic models adjusted for age at death and years of American football participation; AT8 tau pathology and CD68 cell density are stratified into quartiles and OR is for a one quartile increase; PSD-95 concentration is in standardized units; *OR* = odds ratio; B = standardized beta; *n* = 81 (AT8), 84 (CD68), or 37 (PSD-95). Cases were not included for analysis if staining was not successful due to poor tissue quality, the tissue sources had been exhausted, or brains arrived as fragments and did not include the area of analysis

**Table 3:** Binary logistic regression model predicting TDP-43 pathology and dementia

	TDP-43 pathology				Dementia	
	OR	95% CI	*p*-value	OR	95% CI	*p*-value
*TMEM106B* minor G allele (additive)	0.89	0.39–2.57	0.77	0.40	0.16–0.99	0.05

*TMEM106B* genotype *rs3173615*; additive genetic model adjusted for age at death and years of American football participation; *n* = 86

## Discussion

In a series of former American football players with neuropathologically confirmed CTE, we found that rs3173615, a coding SNP in *TMEM106B* that was previously implicated with risk of FLTD-TDP, was associated with p-tau density, CD68 density, synaptic loss, and dementia status in case-only analyses. However, this variant was not associated with risk of CTE in an analysis that compared allele and genotype frequencies between the CTE cases and a group of neurologically normal controls.

Although several genes have been proposed as potential CTE risk factors, this is the first study to demonstrate that a variant in *TMEM106B* is associated with CTE-related outcomes. The findings in this study provide further evidence that variation in *TMEM106B* is linked with neurodegeneration. Interestingly, the effects of *TMEM106B* are heterogeneous across all diseases with which it has been associated. In FTLD, hippocampal sclerosis, and *APOE ϵ4*-negative AD, variation in *TMEM106B* is associated with disease risk [14, 28, 35, 43]. However, variation in *TMEM106B* does not change the risk for ALS, but among those with ALS, it is associated with the presentation of cognitive impairment [44]. Similarly, in the present study, the *TMEM106B* SNP rs3173615 was not associated with CTE risk, but among those with CTE, it modified the neuropathological and clinical presentation, influencing p-tau density, CD68 density, synaptic loss, and odds of dementia.

Rs3173615 underlies the production of two different isoforms of the TMEM106B protein. The more common C allele codes for a highly conserved threonine (T185), whereas the less frequent G allele codes for a substitution of serine (S185) at this location [30]. Functionally, the protein product with S185 is more rapidly degraded than the protein product with T185 [30]. This results in more TMEM106B protein present in C allele carriers. Although the full mechanistic action of the protein is unclear, TMEM106B has been implicated in controlling the size, shape, and acidification of the lysosome [5]. Overexpression of TMEM106B can result in larger lysosomes that do not properly acidify and have impaired function. Enhanced TMEM106B expression has also been demonstrated to result in cell oxidative stress and cytotoxicity [41]. These changes present in rs3173615 G allele carriers with CTE could help explain the increase in microglial activation and p-tau when compared to those lacking this allele. A properly functioning lysosome is critical for the phagocytosis and elimination of toxic proteins. Microglia as well as neurons have been shown to participate in the removal of proteins such as Aβ [8] and tau [2, 33]. An impaired lysosome could result in the buildup of pathogenic proteins as seen in CTE. Future studies will be needed to investigate impaired lysosomal function and how that might prevent proper elimination of ptau.

The association of *TMEM106B* with neuroinflammation as indicated by increased CD68-positive cell density in participants with CTE is consistent with a recent GWAS of age-associated transcriptional changes that identified a genome-wide significant finding with *TMEM106B*. The *TMEM106B* signal was amplified when the outcome was limited to a microglia-associated gene expression cluster [34]. Furthermore, ex vivo analysis in monocyte-derived dendritic cells showed an enhanced stimulated inflammatory response in participants with the risk allele [34]. This increased sensitization is similar to the priming response observed in microglia [31]. Microglia that have been exposed to a previous inflammatory stimulus can exhibit enhanced reactive markers and a more severe immune response when stimulated for a second time [32]. Microglia from individuals with the risk allele might exist in a primed-like state. Exposure to RHI may thus elicit an increased glial reactivity. Our previous work on CTE suggests that microglia and p-tau exist in a positive feedback loop where each component can enhance the other [10]. However, the full spectrum of microglial-mediated inflammatory changes will not be fully captured using only one marker (CD68). Future studies should utilize a combination of histologic markers and biochemical techniques to further explore the effect *TMEM106B* on neuroinflammation.

Finally, *TMEM106B* genotype was related to dementia status, adjusting for age at death and duration of football play. For each protective minor allele, odds of dementia decreased by 60%. This finding provides insight into why certain individuals with CTE may progress to dementia while others have slower progression and do not become functionally impaired, even though the exposure to contact sports might be similar. One possible pathologic mechanism for the *TMEM106B*-dementia relationship comes from the relationship between *TMEM106B* and PSD-95 protein levels. PSD proteins are post-synaptic density proteins that are often used as markers of synaptic loss and dysfunction [13]. Loss of synaptic proteins can impair neuronal signaling and recruitment of essential neuronal proteins, leading to loss of long-term potentiation and cognitive dysfunction. Synapse loss can occur through a variety of ways [37]. Two common pathways for synaptic loss that may occur in CTE are neuronal death and microglia-mediated synaptic pruning [11]. These pathways may be mediated by tau-induced synaptic dysfunction and neuroinflammation respectively. Future studies should further tease apart the mechanisms leading to synaptic loss and its relationship to clinical impairment.

There are several limitations to this study. CTE cases were largely self-selected or referred by the next-of-kin after death and are not necessarily representative of all individuals who play football. However, selection should only bias a genetic relationship if there are pleiotropic effects that influence selection into the study [25]. Additionally, methods for determination of RHI exposure and clinical and medical history depended on retrospective review and inaccuracies associated with informant-report may introduce measurement error. Another limitation comes from the inclusion of a separately genotyped control group for the case-control analysis. As the control group was clinically, but not neuropathologically assessed, it is possible they may have underlying sub-clinical pathology. Additionally, although the same genotyping platform was used for cases and controls, they were genotyped in separate batches, potentially introducing bias. Ideal controls would have played football and would not have evidence of CTE or other neurodegenerative pathology. Unfortunately, most football players from the VA-BU-CLF brain bank have evidence of CTE pathology; therefore, we relied on controls from another study who may have developed CTE if they were exposed to football. This misclassification may have biased our case-control analysis toward the null, but would not affect our case-only analyses. Future studies should include controls with a complete athletic history and neuropathological evaluation and should not genotype cases and controls separately. An additional limitation is the small sample size by genetic standards. However, studies have only recently ascertained contact sport history or conducted neuropathological examinations for CTE. The current study was conducted in the largest group of CTE cases available to date. Additionally, to maximize statistical power, these cases were densely phenotyped using a quantitative measure of tau pathology. Nonetheless, the findings should be interpreted with caution until they can be independently replicated. Lastly, sufficient genetic data was not available to account for population substructure, which could confound a genetic relationship. However, the analysis was limited to informant reported Caucasian participants to grossly account for population differences. Future studies will be needed to better understand the effects of *rs3173615* in non-Caucasian ethnicities.

## Conclusions

In conclusion, this study reports one of the first genetic associations for CTE-related outcomes. Although *TMEM106B* was not associated with CTE case-control status, in case-only analyses, the minor allele had a protective effect for multiple CTE-related neuropathological outcomes including neuroinflammation, p-tau density and synaptic dysfunction. Similarly, in case-only analyses, the minor allele had a protective effect for dementia. Future work is required to replicate these findings in an independent sample and to determine the mechanism by which *TMEM106B* interacts with RHI and other genetic risk factors to modify CTE-related outcomes. Overall, *TMEM106B* genotype may partially explain why some individuals experience more severe CTE- related outcomes while others are spared despite similar exposure to contact sports.
